# History of the Indiana State Dental Association—1858 to 1882

**Published:** 1883-02

**Authors:** 


History of the Indiana State Dental Association, 1858 to 1882. Also transactions of the twenty-fourth annual session from June 27th, 28th and 29th, 1882. This volume is just at hand, and is interesting, not only in the presentation of the transactions of the last meeting, which was a very good one indeed, but especially for the historical account of one of the oldest and best of our *State* Dental Societies. This was one of the first State organizations formed. It was one of the first, and I think the first to introduce clinics during its sessions. Its meetings have always been well sustained, and it has been the means of great good for the profession, not only of Indiana, but for that of the adjacent States as well, as from these there have always been members of the profession present, who were granted privileges in the work of the meetings. The organization and establishment of the Indiana Dental College, was effected through the agency of the State Society. This volume may be obtained of Dr. W. M. Herriott, of Indianapolis.

